# Clinical study for external Chinese herbal medicine LC09 treating hand-foot skin reaction associated with the antitumor targeted drugs: Protocol for a prospective, randomized, controlled, double-blind, and monocentric clinical trial

**DOI:** 10.1097/MD.0000000000018849

**Published:** 2020-01-24

**Authors:** Gui Wang, Liqun Jia, Yuying Pei, Ran Yu, Yu Gao, Chao Deng, Yanni Lou

**Affiliations:** a Department of Oncology of Integrative Chinese and Western Medicine, China-Japan Friendship Hospital; b Beijing University of Chinese Medicine, Beijing, China.

**Keywords:** Chinese herbal medicine, hand-foot skin reaction, LC09, molecular targeted anticancer drugs, randomized controlled trial

## Abstract

**Background:**

Molecular targeted anticancer drugs such as multikinase inhibitors have shown obvious therapeutic advantages in a variety of tumors. The occurrence of hand-foot skin reaction (HFSR) is positively correlated with therapeutic effect, but it is also the most common cause of dose limiting toxicity for this treatment. This can lead to interruption or decrement of the treatment, a reduction in quality of life for patients, as well as potentially leading to secondary infections. As a result, the curative effect of targeted anticancer drugs will be negatively impacted. Currently, there is no certain and effective therapy. External use of Chinese herb medicine LC09 in the early treatment of HFSR has shown positive outcomes, but it is necessary to carry out further clinical research to confirm.

**Objectives:**

The purpose of this study was to investigate the efficacy and safety of topical soaks of Chinese herbal medicine LC09 for HFSR induced by molecular targeted anticancer drugs.

**Methods:**

The trial is a prospective, randomized, controlled, double-blind, monocentric, and interventional study. A total of 66 patients with HFSR will be recruited and randomly assigned to receive either LC09 Granules or placebo. The primary outcomes are the assessment of HFSR grade and pain score. The secondary outcomes are the evaluation of the quality of life, incidence of targeted drug dosage reduction, and incidence of targeted drug withdrawal.

**Discussion:**

This prospective, randomized clinical trial will provide valuable data regarding the efficacy and safety of topical soak treatments with LC09 granules for HFSR. Positive results would provide evidence-based complementary therapeutic approach future treatments of HFSR.

**Trial registration:**

Chinese Clinical Trial Registry, http://www.chictr.org.cn, ChiCTR1900023679. Registered on 7 June 2019.

## Abstract 2

Supplemental Digital Content is available in the text

## 1 Introduction

The molecular targeted anticancer drugs with high selectivity have recently been receiving more and more attention. These drugs provide patients with promising long-term survival by blocking specific molecular targets, which in turn inhibit the growth, development, and spread of cancer cells. So far, there are 2 main types of targeted therapy drugs: monoclonal antibodies that target extracellular regions of surface receptors, in addition to tyrosine and serine threonine kinase inhibitors.^[1]^ Targeted therapy, which can disrupt the signal transduction of tumor cells and inhibit the proliferation of tumor cells, is both more efficient and safer than conventional cytotoxic chemotherapy drugs. Currently, more than 20 kinase inhibitors are approved by the FDA in the United States, for the treatment of various advanced cancers resistant to conventional chemotherapy. These have proven clinical success.^[2]^ However, these drugs can produce significantly adverse skin reactions. Hand-foot skin reaction (HFSR) is the most clinically significant adverse event after targeted drug therapy, which seriously affects patients’ quality of life. As well, it may lead to drug dose reduction or forced interruption of treatment, thereby affecting any potential benefits resulting from the treatment. The incidence and severity of HFSR is determined by the types and doses of targeted anticancer drugs used in treatments. Of the available multikinase inhibitor (MKI) drugs, the incidence of HSFR induced by sorafenib, regorafenib, sunitinib, and cediranib was 10% to 62%, 60.5%, 10% to 50%, and 35.5% respectively.^[3–6]^ In addition, BRAF inhibitors (vemurafenib, dearfenib) and epidermal growth factor receptor inhibitors have also been reported to cause HSFR.^[7–9]^ The incidence of HSFR induced by vermorafenib was reported to be more than 50%.^[10]^

The clinical appearance of HFSR is similar to hand-foot syndrome caused by cytotoxic drugs, but with its own characteristics. HFSR symptoms typically appear within the first 2 to 4 weeks of initiating targeted anticancer therapy, which are characterized by: tingling, burning, painful sensations in the palms and soles, symmetrical erythema, tense bullae, focal hyperkeratosis, and decreased tolerance to contact with objects.^[11]^ The above symptoms often occur at the same time or one after another, where the stress-bearing areas of the hands and feet often exhibit more severe symptoms.^[12]^ The precise pathogenesis of HFSR is not completely understood. However, it is thought that MKI inhibition of various targeted receptors that are expressed in normal tissues, may be the main mechanism.

There has been exploratory observation of therapeutic drugs, which includes urea ointment, ceramide hydroglia, and oral celecoxib (of which both are preventive studies).^[13,14]^ The results show that the incidence of HFSR can be reduced and the even delayed at the time of occurrence, but that targeted drug reduction and withdrawal still remain the best available option for treatments.^[1,15,16]^ Therefore, it is particularly important to find an effective method for the prevention and treatment HFSR in the era when target drugs are more and more widely used.

Chinese herb medicine (CHM) has a long history and advantages in treating dermatologic disorders^[17]^ and cancer.^[18]^ This is where CHM external treatment may have characteristics and advantages as a potential treatment for HFSR. There were many attempts to treat targeted anticancer therapies-related dermatologic toxicities with traditional Chinese medicine. Bos et al^[19]^ reported an improvement in HFSR of 4 patients after being treated by topical psoralen plus ultraviolet (UV)-A therapy. Zhao et al^[20]^ investigated the effectiveness of Tao-hong-si-wu (TCM) in HFSR and HFS by a randomized controlled trials (RCT), and the results revealed that it had a higher effective rate than oral pyridoxine, as well as a significant improvement in HSFR and quality of life, such as pain relief, improvement of daily life, and walking ability. Tian et al^[21]^ conducted a RCT to investigate the efficacy of topical application of compound Danxiong granules for treatment of dermatologic toxicities associated with targeted anticancer therapies, and it revealed topical application of Danxiong granules could effectively attenuate dermatologic toxicities induced by targeted anticancer therapies, and the effect of Danxiong granules was more pronounced in HFSR. LC09 consists of 5 herbal granules, namely Astragali Radix (Huangqi), Angelicae Sinensis Radix (Danggui), Erodii Herba Geranii Herba (Laoguancao), Arnebiae Radix (Zicao), and Carthami Flos (Honghua), and has been used to treat HFSR in clinics for many years with good effects. However, there is a lack of large-sample, well-designed double-blind, placebo-controlled trials to support the evidence. Therefore, the efficacy and safety of LC09 for HFSR treatment is worthy of a scientific randomized controlled trial. In the present study, we assumed that LC09 is efficacious with respect to the alleviation of the signs and symptoms of HFSR. If successful, the study may provide an evidence-based complementary therapeutic approach to slow or prevent the clinical progression of HFSR in patients taking molecular targeted anticancer drugs. In this protocol, we detail the overall study design and approach.

## 2 Methods

### 2.1 Trial objective

This research will evaluate the efficacy and safety of a topical soak of Chinese herbal medicine LC09 for HFSR induced by molecular targeted anticancer drugs. It is hoped this will clarify the role of LC09 in the clinical medical field as a treatment for HFSR. We will evaluate the effectiveness of LC09 by comparing the changes in HFSR grading and pain score at 1 week after randomization between the LC09 group and the placebo group. Adverse events (AEs) that occur during the study will be investigated to evaluate the safety of LC09.

### 2.2 Study design

**Figure 1:**
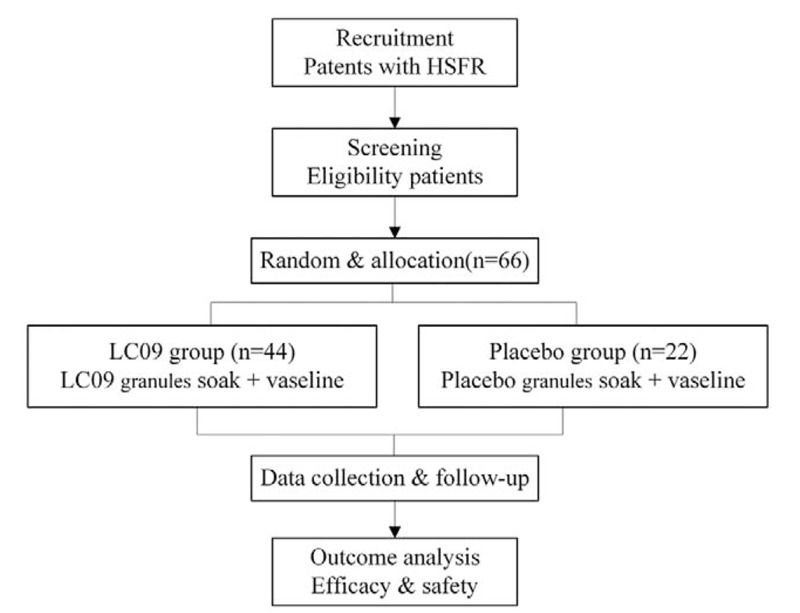
Flow chart of the clinical study.

This study is a single-center, randomized, double-blind, placebo-controlled, prospective, clinical study. The trial has been approved by the Ethics Committee of Clinical Research of China-Japan Friendship Hospital (2018-81-K56) and registered at Chinese Clinical Trial Registry (ChiCTR1900023679). Trained researchers will introduce the trial to patients, giving them information sheets and a consent form. All patients must submit written informed consent prior to registration. This study protocol conforms to the Standard Protocol Items: Recommendations for Interventional Trials guidelines. We will rigorously follow the Consolidated Standards of Reporting Trials Extension for Chinese Herbal Medicine Formulas 2017 recommendations in reporting the results. In this study, a total of 66 patients with HFSR will be recruited from Integrative Chinese and western medicine oncology departments of the China-Japan Friendship Hospital. The flow chart of this trial is shown in Fig. 1.

### 2.3 Eligibility criteria

Inclusion criteria

1. Patients with malignant tumor diagnosed by evidence of pathological or cytological findings.
2. HFSR above grade 1, after the treatment with targeted anticancer drugs.
3. Aged between 18 and 80 years old.
4. ECOG score is 0 and 2 points.
5. A life expectancy of at least 3 months.
6. No major organ dysfunction, heart, liver, kidney function is basically normal, and test indicators meet the following requirements: Neutrophil > 1.5 × 10^9^/L, platelet > 100 × 10^9^/L, hemoglobin > 90 g/L. Bilirubin < 1.5 × ULN; AST, ALT < 2.5 × ULN; Serum creatinine < 1.5 × ULN; Endogenous creatinine clearance (Ccr) ≥60 mL/min (Cockcroft-Gault formula).
7. Those who can cooperate with the assessment of HFS classification, agreed to participate in this study and sign informed consent forms.

Exclusion criteria:

1. HFSR combined with the presence of other skin lesions in other extremities (such as peripheral neuropathy caused by diabetes or chemotherapy, hand and foot syndrome, hand and foot fungal infection, skin trauma).
2. Other drugs that may affect HFSR that have been planned to be applied (including urea cream, vitamin B6, etc).
3. Patients with severe dysfunction of the heart, lung, liver, or kidneys, infections, or other serious disease unable to tolerate anticancer therapy.
4. Pregnant or lactating women.
5. Participants in other clinical trials at present or within 4 weeks.
6. Intellectual or mental disorders that may affect comprehension and informed consent.

### 2.4 Sample size calculation

In previous studies, the effective rate of HFSR treated with LC09 was 85.71% (12/14), which was set as p1. The effective rate of placebo group was 46.15% (6/13), which was set as p2. Conditions were set as α=0.05, β=0.10, for a 2-sided test. Based on 2 population rate hypothesis tests designed completely randomly, the ratio of LC09 group and placebo group is 2:1. By applying the sample size formula, it was calculated that the sample size should be 17 cases in the placebo group. Considering that the dropout rate was 20% during the trial, the required sample size came to 21.25 cases (22 cases) in the placebo group. Therefore, there were 44 cases in LC09 group, 66 cases in total.

### 2.5 Randomization, allocation concealment, and blinding

A stratified and block randomization design will be adopted. The distribution ratio of the LC09 group and placebo group will be 2:1, and the block size 6. The block randomization sequence will be generated by SPSS software and saved in a sealed envelope by an independent clinical statistician. Eligible participants will be randomly allocated to either the LC09 group or the placebo group and they will receive corresponding drugs based on their random number. The randomization design is provided by the Beijing Guoxin Ze Ding Technology Co, Ltd.

The participants, researchers, and statisticians will be blinded to group allocation. Randomized lists and blind codes will be kept strictly confidential in the third statistical department, until the entire program is complete (except to individual patients in emergency situations).

### 2.6 Intervention

The test drugs are LCO9 granules and LC09 mimetic agent, provided by pharmacy department of the China-Japan Friendship Hospital. The composition of specific Chinese herbal remedies is summarized in Table 1. The herbs in the prescription will be mixed, cooked, filtered, and pressure spray-dried to form granules. The preparation of the placebo includes adding artificial pigment to the particle excipient to make an imitation drug, which will be matched as closely as possible in appearance and taste to the real granules. The granules will be packaged into small single-dose sachets, each weighing 16 g. All the drugs will be uniformly packaged and identified with the same labels.

**Table 1:**
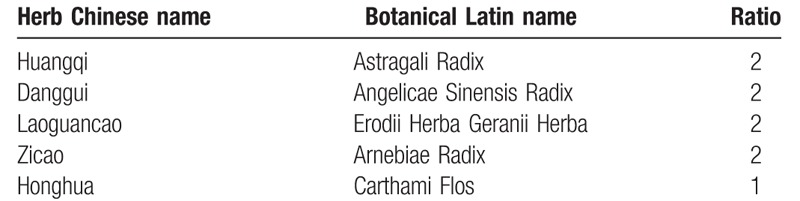
Constituents of LC09 formula.

Administration method: 32 g of the granules will be dissolved in 60°C to 70°C water and diluted to 1000 mL in thermostatic foot bath tub with herbal liquid temperature 35°C to 38°C. This is then used to soak the hands and feet for 20 minutes twice a day. After washing and immersion intervention, patients in both groups are to apply vaseline moisturizer on their hands and feet for local moisturizing. Treatment duration lasts for 7 consecutive days.

### 2.7 Outcomes

Primary outcomes.

1. HFSR grading
  HFSR grading will be recorded daily through patient records and consultation. HFSR is graded as 3 levels according to National Cancer Institute (NCI-CTCAE) Version 4.03:^[22]^ Minimal skin changes (e.g., erythema, swelling, or hyperkeratosis without pain). Skin changes (e.g., blistering, bleeding, edema, or hyperkeratosis) with pain; limiting instrumental activities of daily living. Severe skin changes (e.g., blistering, bleeding, edema, or hyperkeratosis) with pain and limiting self-care activities of daily living.
  Evaluation of effect: “Cure” is indicated by the absence of skin lesions. “Effective” is defined as a reduction in at least 1 grade in HSFR grading. “Ineffective” is defined as no improvement in HSFR grading. The total effective rate is then defined as the number of patients who achieved “cure” and “effective” divided by the total number of patients.
2. Pain score
  Adopting numerical rating scale (NRS) of pain criteria is to be used as the main evaluation. NRS score is to be recorded daily by both patient diary and consultation, where a scale from 0 to 10 points represents the patient's pain level. The specific division are as follows: painless (0 points), mild pain (1–3 points), moderate pain (3–6 points), severe pain (6–9 points), and very severe pain (9–10 points). Table 2 shows the degree of pain relief. Pain relief rate (RR) = CR+ PR+ MR.

**Table 2:**
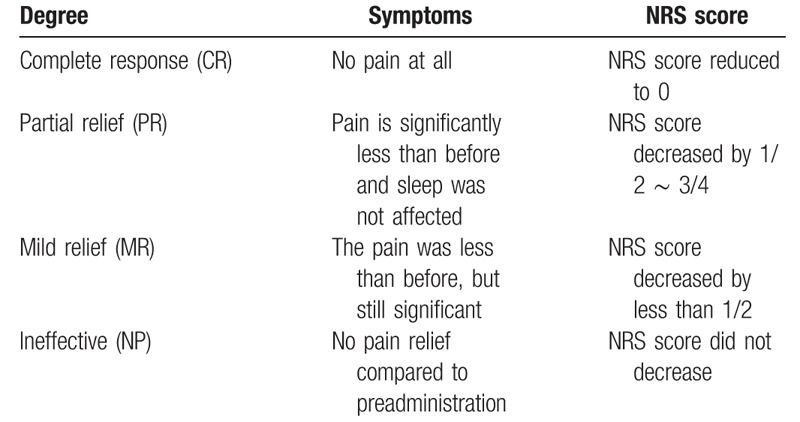
Pain relief.

Secondary outcomes.

1. Quality of life (QOL)
  The validity and reliability of HF-QoL has been evaluated by different researchers.^[23,24]^ The HF-QoL is composed of a 20-items local symptom evaluation and 18-items daily activity scale, see supplemental digital content (appendix 1).^[23]^ The researchers will explain what each item means and how to complete the questionnaire before giving it to the participants. Patients are required to complete the questionnaire by themselves after coming to fully understand it. Researchers will then collect the questionnaires immediately after patients complete them.
2. Targeted drug dosage reduction incidence (%) = the number of cases of targeted drug reduction caused by HFSR in each group/the total number of cases in each group × 100%.
3. Targeted drug withdrawal incidence (%) = the number of cases of targeted drug withdrawal caused by HFSR in each group/the total number of cases in each group × 100%.

### 2.8 Safety assessments

1. Skin irritation response score
  Erythema and eschar: 0 for no erythema, 1 for slight erythema (barely visible), 2 for moderate erythema, 3 for severe erythema, 4 for moderate erythema to mild eschar formation; Edema: 0 points for no edema, 1 point for slight edema (barely visible), and 2 points for slight edema (Clear outline of uplift), 3 points for moderate edema (swelling of about 1 mm), and 4 points for severe edema (swelling of more than 1 mm). Mean reaction score = (total score for erythema formation + total score for edema formation) /total score. A mean reaction score < 0.5 was defined as no irritation.
2. Skin allergy score
  The scoring criteria and calculation methods were the same as skin irritation scores.
3. Laboratory examination
  A blood routine examination, routine urine test, liver function test, renal function test, coagulation function test, tumor marker, and electrocardiograph will be carried out for safety outcomes, which will be monitored at enrollment and after 7 days of treatment.

### 2.9 Study end points

By analyzing and summarizing the clinical observation data of a small sample in the early stage, it was concluded that the effective time of TCM external treatment is generally within 7 days. Therefore, the endpoint event of the study is defined as:

1. Seven days from external intervention using CHM.
2. If HFSR is not relieved or aggravated after TCM intervention.
3. If intolerable side effects caused by external agentia arise, judged by a physician.

### 2.10 Data collection and management

Data collection schedule is presented in Table 3. Data collection from the day before treatment to the end of treatment will be recorded on the predesigned case report form and entered into Epidata v3.1 for management. All private and sensitive data will be deleted and all information about the patient's identity will be encoded.

**Table 3:**
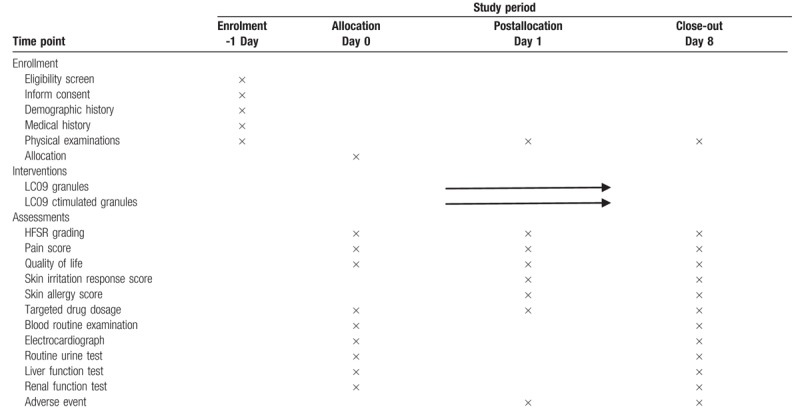
Schedule for the treatment and the outcome measurements.

### 2.11 Statistical analysis

All statistical analyses will be performed with SPSS20.0 software (SPSS Inc, Chicago, IL). Continuous data will be presented as mean ± standard error. Categorical data will be expressed as frequencies and percentages. A paired *t* test will be used to compare before and after treatments within 1 group. To compare the difference between 2 groups, independent *t* tests (or a Mann–Whitney *U* test) will be used to analyze continuous data. The *χ*^2^ or Fisher exact test will be used for categorical data. The primary and secondary indicators will be analyzed by a full analysis set and per protocol set. All statistical tests will be 2-tailed and *P < *.05 will be considered statistically significant difference.

### 2.12 Adverse events

All AEs will be documented and any serious adverse events will be reported to the research ethics committee within 24 hours. Where AEs occur, the investigator will ask the patient to terminate the topical soak treatment and determine if the event is related to the study formula. The lead investigator can implement emergency safety measures to protect patients from direct harm. If the AE is related to the study formula, the severity of the AE is evaluated to determine whether the patient can continue to participate in the study. If a serious adverse event is observed, the patient will drop out of the study.

### 2.13 Withdrawal and dropout

We will monitor patients for AEs during the study period and record any incidents in the case report forms. The trial will be discontinued on the condition that any serious adverse events happen. Patients may request to be withdrawn from this study at any time without any reason. The researchers will record the reason for any interruption in the intervention and whether each participant completed the study.

### 2.14 Ethics and dissemination

This trial will be conducted in accordance with the latest revision of the Declaration of Helsinki governing standards for good clinical practice. Participants will voluntarily sign a written informed consent form before joining the study and can withdraw from the study at any time for any reason. Patient confidentiality will be guaranteed as the data will be deidentified. The results of the clinical trial will then be published independently and transparently, regard less of the results.

## 3 Discussion

As the most clinically significant and dose-limiting adverse event of targeted anticancer drugs, HFSR limits the tolerance and compliance of tumor patients receiving targeted drug therapy. Though not life threatening, HFSR severely impacts quality of life and potentially limits the anticancer effect. The incidence of HFSR with regorafenib was found to be up to 60.5% and the incidence of severe HFSR up to 20.4%.^[25]^ Evidence has also shown that Asians are more susceptible than Caucasians to HFSR, which is associated with certain MKIs, such as sorafenib, sunitinib, and pazopanib.^[26]^ However, to date, no prevention or treatment strategy has proven incontrovertibly effective. With more and more targeted anticancer drugs being widely used, finding effective methods to prevent and treat HFSR is essential and will enable patients to receive the promising therapies.

CHMs have been considered for HFSR treatment due to their lack of side effects and excellent efficacy. LCO9 is composed of 5 herbs, which can invigorate energy, activate blood circulation, detoxify and generate muscle. Early small sample observation test found that LC09 could alleviate symptoms and downgraded disease severity of HSFR, and most of the improvement occurred within 7 days of taking the drug. However, there are no rigorously designed clinical trials for evaluating the efficacy and safety of LC09 for HFSR induced by molecular targeted anticancer drugs. Thus, we propose this double-blind, randomized controlled study to assess the efficacy and safety of a topical soak with LC09 for HFSR.

A suitable control group is essential for designing high-quality clinical trial. The study will use granules of a similar color and taste as placebo controls to ensure blinding, so that the results of this trial provide high-quality evidence to the efficacy of CHM in the treatment of HFSR. Topical application is the most accessible type of herbal treatment. Topically applied herbal lotion can directly work on the focus of lesion and this way reduces the first-pass effect through the liver and gastrointestinal degradation. Most commonly used is a herbal soak, which is an alternative therapy usually applied for HFSR treatment. Intelligent thermostatic foot bath apparatuses are strictly adopted in this study to ensure the consistency of intervention conditions and avoid bias. The results of the RCT designed trails will provide valuable data for confirming the effectiveness and safety of LC09.

## 4 Trial status

Recruitment for the trial started in June 2019 and is ongoing.

## Author contributions

**Conceptualization:** Gui wang, Yanni Lou.

**Data curation:** Yuying Pei, Gui Wang, Ran Yu, Yu Gao.

**Formal analysis:** Liqun Jia.

**Investigation:** Yuying Pei, Ran Yu, Yu Gao, Chao Deng.

**Project administration:** Yanni Lou, Liqun Jia.

**Supervision:** Liqun Jia, Yanni Lou.

**Writing – original draft:** Gui wang.

**Writing – review & editing:** Chao Deng, Yanni Lou, Liqun Jia.

## Supplementary Material

Supplemental Digital Content
