# Supplementary material for: Clinical study for external Chinese herbal medicine LC09 treating hand-foot skin reaction associated with the antitumor targeted drugs: Protocol for a prospective, randomized, controlled, double-blind, and monocentric clinical trial
Source: Medicine (Baltimore). 2020 Jan 24;99(4):e18849. doi: 10.1097/MD.0000000000018849 (PMC7004671; doi:10.1097/MD.0000000000018849)
Supplement: Supplemental Digital Content [file medi-99-e18849-s001.pdf]

Appendix : Revised HF-QoL1.0

Hand-Foot reaction and Quality of Life questionnaire (HF-QoL)   Page 1

Dear Participant: Thank you for agreeing to complete this survey.

Having cancer, and receiving treatment for it may affect certain aspects of a patient's life. Because some cancer medications may cause side effects on the hands, feet, or other body areas in some patients, we would like to know to what extent your treatment has affected you.

Please complete each question below regardless of whether or not you have had any skin reactions on your hands, feet or other areas of the body.

Your responses will be kept strictly confidential and will not be shared with your doctor by the study team.

Please turn to the next page

***Your Feet*:**

For each question below, please circle the number that best describes your experience with your feet over the past week. (If you did not have any problems with your feet, please answer each item accordingly).

During the LAST WEEK…

	**To what extent did your feet:**	**Not at all**	**A little**	**Somewhat**	**Very much**	**Extremely**
1.	*Have red or discolored skin?*	0	1	2	3	4
2.	*Show changes in color or appearance of toenails?*	0	1	2	3	4
3.	*Feel painful?*	0	1	2	3	4
4.	*Feel sensitive to pressure or walking on hard surfaces?*	0	1	2	3	4
5.	*Cause you a burning or "hot" sensation?*	0	1	2	3	4
6.	*Have cracked or peeling skin?*	0	1	2	3	4
7.	*Have thickened or calloused skin?*	0	1	2	3	4
8.	*Feel numbness or tingling?*	0	1	2	3	4
9.	*Feel swollen?*	0	1	2	3	4
10.	*Have blisters or sores?*	0	1	2	3	4

Please turn to the next page

Your Hands or Fingers:

For each question below, please circle the number that best describes your experience with your hands or fingers over the past week. (If you did not have any problems with your hands or fingers, please answer each item accordingly).

During the LAST WEEK…

	To what extent did your hands or fingers:	***Not at all***	***A little***	***Somewhat***	***Very much***	***Extremely***
11	*Have red or discolored skin?*	*0*	*1*	*2*	*3*	*4*
12.	*Show changes in color or appearance of fingernails?*	*0*	*1*	*2*	*3*	*4*
13.	*Feel painful?*	*0*	*1*	*2*	*3*	*4*
14.	*Feel sensitive to pressure?*	*0*	*1*	*2*	*3*	*4*
15.	*Cause you a burning or "hot" sensation?*	*0*	*1*	*2*	*3*	*4*
16.	*Have peeling skin?*	*0*	*1*	*2*	*3*	*4*
17.	*Have thickened or calloused skin?*	*0*	*1*	*2*	*3*	*4*
18.	*Feel numbness or tingling?*	*0*	*1*	*2*	*3*	*4*
19.	*Feel swollen?*	*0*	*1*	*2*	*3*	*4*
20.	*Have blisters or sores?*	*0*	*1*	*2*	*3*	*4*

Please turn to the next page

Your Daily Activities:

For each question below, please circle the number that best describes your experience with your daily activities over the past week. (If you did not have any problems with your daily activities due to skin reactions, please answer each item accordingly).

During the LAST WEEK…

	How often did your skin reaction cause you to…	***Not at all***	***A little***	***Sometimes***	***Often***	***Always***
21.	*Avoid physical activity vigorous enough to make you sweat?*	*0*	*1*	*2*	*3*	*4*
22.	*Have difficulty doing heavy housework?*	*0*	*1*	*2*	*3*	*4*
23.	*Walk at a slower pace than usual?*	*0*	*1*	*2*	*3*	*4*
24	*Have difficulty going up or down stairs?*	*0*	*1*	*2*	*3*	*4*
25.	*Have difficulty lifting or carrying something heavy, such as a bag of groceries?*	*0*	*1*	*2*	*3*	*4*
26.	*Have difficulty using your fingers – such as buttoning a shirt or picking something up?*	*0*	*1*	*2*	*3*	*4*
27.	*Accomplish less than you had liked at home or at work?*	*0*	*1*	*2*	*3*	*4*
28.	*Have difficulty eating with a fork or knife, or drinking from a glass?*	*0*	*1*	*2*	*3*	*4*
29.	*Have difficulty with bathing or cleaning yourself?*	*0*	*1*	*2*	*3*	*4*
30.	*Have to wear different clothes or shoes than usual?*	*0*	*1*	*2*	*3*	*4*
31.	*Have to stay off your feet all or most of the day?*	*0*	*1*	*2*	*3*	*4*

Please turn to the next page

Your Social Life:

For each question below, please circle the number that best describes your social life over the past week. (If you did not have any problems with your social life due to skin reactions, please answer each item accordingly).

During the LAST WEEK…

	**How often did your skin reaction cause you to:**	**Not at all**	**A little**	**Sometimes**	**Often**	**Always**
32.	*Feel less comfortable than usual in social activities?*	0	1	2	3	4
33.	*Feel isolated from others?*	0	1	2	3	4
34.	*Cut back on your social activities?*	0	1	2	3	4

Your Feelings or Mood:

For each question below, please circle the number that best describes your feelings or mood over the past week. (If you did not have any problems with your feelings or mood due to skin reactions, please answer each item accordingly).

During the LAST WEEK…

	**How often has your skin reaction caused you to feel:**	**Not at all**	**A little**	**Sometimes**	**Often**	**Always**
35.	*Less confident that you could overcome problems?*	0	1	2	3	4
36.	*Bothered by things that usually don't bother you?*	0	1	2	3	4
37.	*Helpless?*	0	1	2	3	4
38.	*Downhearted and sad?*	0	1	2	3	4

Thank you for completing this questionnaire.

Copyright (c) 2007, 2008 by Bayer Pharmaceuticals Corporation. All rights reserved.
